# The prevalence of nonalcoholic fatty liver disease in people living with HIV: a systematic review and meta-analysis

**DOI:** 10.1186/s12879-025-10455-y

**Published:** 2025-02-19

**Authors:** Jiaqi Wei, Wei Hui, Yuan Fang, Han Jia, Yu Yang, Tong Zhang, Hao Wu, Bin Su, Taiyi Jiang

**Affiliations:** 1 Beijing Key Laboratory for HIV/AIDS Research, Sino-French Joint Laboratory for HIV/AIDS Research, Clinical and Research Center for Infectious Diseases, Beijing Youan Hospital, Capital Medical University, Beijing, 100069 China; 2 Beijing Youan Hospital, Telemedicine and Connected Health Center, Capital Medical University, Beijing, 100069 China

**Keywords:** HIV, NAFLD, Liver fibrosis, Meta-analysis

## Abstract

**Background:**

Owing to long-term antiretroviral therapy (ART), the incidence of non-HIV-related chronic diseases is increasing, and liver disease is the leading cause of increased AIDS mortality. Moreover, the prevalence of NAFLD and liver fibrosis has been reported to vary widely across regions and studies. There is no precise description of the trend and characteristics of NAFLD in PLWH. Here, we aimed to explore the prevalence and outcomes of NAFLD in people living with HIV (PLWH).

**Methods:**

The PubMed, Web of Science, Embase, and Cochrane Library databases were searched on August 15, 2023, for studies that evaluated the prevalence of NAFLD or liver fibrosis among PLWH. The meta-synthesized effects of NAFLD and liver fibrosis were the primary outcomes, and potential moderators were the secondary outcomes. The meta-analysis of the combined event rate (ER) and random effects was conducted on the basis of the number of individuals with NAFLD, the number of individuals with liver fibrosis, and the total sample size.

**Results:**

Of the 3520 studies identified, 41 studies were eligible for the meta-analysis. The results revealed that the combined ERs of NAFLD and liver fibrosis were 0.38 (95% CI: 0.33–0.43, *p* < 0.01) and 0.25 (95% CI: 0.18–0.32, *p* < 0.01), respectively.

**Conclusions:**

This meta-analysis provided empirical evidence that the prevalence of NAFLD and liver fibrosis in PLWH is greater than that in the general population, which requires sufficient attention. In the HIV population, noninvasive imaging to monitor NAFLD changes should be strengthened, and a high TG level might be an early predictive indicator for HIV-associated fatty liver disease; however, large-scale prospective clinical research data are still needed for further validation and evaluation.

**Supplementary Information:**

The online version contains supplementary material available at 10.1186/s12879-025-10455-y.

## Introduction

Acquired immune deficiency syndrome (AIDS), which is caused by human immunodeficiency virus (HIV), is one of the leading global health threats. There are approximately 38.4 million people living with HIV (PLWH) globally, and 650,000 people died from AIDS-related illnesses in 2021 [1]. With the application of highly effective antiretroviral therapy (ART), AIDS has become a controllable (although not curable) chronic infectious disease. Owing to long-term ART treatment, the incidence rates of non-HIV-related chronic diseases, such as cardiovascular disease (CVD) and chronic liver disease (CLD) [2], are increasing, and these diseases are gradually becoming the main causes of AIDS-related mortality [3]. Among these diseases, liver disease is a leading cause. A recent study involving a Data collection on Adverse events of Anti-HIV Drugs (D: A:D) cohort revealed that liver disease accounted for approximately 10% of the deaths among PLWH, making liver disease the third leading cause of non-AIDS-related death in this cohort [3]. The prevalence of nonalcoholic fatty liver disease (NAFLD) is increasing, which has become a new issue that needs attention, especially in the diagnosis and treatment of PLWH [4].

NAFLD is a clinical pathological syndrome characterized by unusual fat storage in liver cells, excluding other liver damage caused by alcohol or other clear reasons [5–7]. NAFLD has led to increased incidence and mortality rates of liver-related and extrahepatic complications. The prevalence of NAFLD in HIV-positive populations varies greatly in different periods because of the application and continuous updating of ART drugs in the clinical setting. In the early ART era, up to 60% of PLWH developed liver damage and microvascular steatosis due to adverse drug reactions, leading to NAFLD [8]. In the modern ART era, although drugs causing severe liver damage are no longer used in treatment, NAFLD is still quite common in the HIV population. The reported prevalence varies widely across regions and studies. Studies have shown that the estimated prevalence of NAFLD in the general population diagnosed by imaging is approximately 29.8% [9], and further stratification has shown that the prevalence can reach as high as 76% and 90% in severely obese patients with diabetes and those who undergo weight loss surgery, respectively [10, 11]. It is predicted that these populations will soon become the main source of patients with liver cirrhosis and hepatocellular carcinoma [12–14]. However, there is no precise description of the trend in the prevalence and characteristics of NAFLD in PLWH.

Considering the wide application of ART drugs, in this study, we aimed to clarify the prevalence of NAFLD and liver fibrosis in the HIV population, identify the most likely risk factors and determine whether these factors can be used as predictive factors for NAFLD, with the aim of providing early intervention measures for the prevention and control of non-HIV-related liver diseases.

## Methods

This work followed the Preferred Reporting Items for Systematic Reviews and Meta-Analysis (PRISMA) guidelines and was registered in the International Prospective Register of Systematic Reviews (PROSPERO; registration No: CRD42022338499; https://www.crd.york.ac.uk/PROSPERO/#recordDetails). The detailed information is available in Supplementary Table 1.

### Search strategy

A comprehensive search was performed in electronic databases, including PubMed, Web of Science, Embase, and the Cochrane Library, from database inception to August 15, 2023, with no limitations on the publication type. The search terms used were intersecting HIV-related terms (human immune deficiency virus OR acquired immune deficiency syndrome OR HIV OR AIDS) and NAFLD-related terms (fatty liver OR nonalcoholic liver OR NAFLD OR NAFL OR NASH OR steatohepatitis). We also searched the reference lists of the selected articles and related review articles to identify missing studies.

## Selection criteria

To be included in this meta-analysis, studies had to report the prevalence of NAFLD or liver fibrosis among PLWH or provide data from which the prevalence of NAFLD or liver fibrosis among PLWH could be calculated. Moreover, studies had to use imaging and/or liver biopsy to diagnose NAFLD or liver fibrosis to be eligible for inclusion. Studies were excluded if they (1) were not associated with HIV; (2) were research protocols or feedback reports; (3) were case reports; (4) were review articles; (5) were child-oriented; or (6) did not provide data from which the prevalence of NAFLD or liver fibrosis among PLWH could be calculated. Duplicate studies were removed *via* EndNote X9 software, and two reviewers (JQW and WH) separately evaluated the search results on the basis of titles and abstracts. The remaining articles were further evaluated by JQW and WH *via* full-text assessment. Disagreements about eligibility between reviewers were resolved by discussion with TYJ and BS.

## Data extraction

Relevant data were independently extracted and crosschecked by JQW and YF *via* an Excel spreadsheet. The outcomes of interest were the prevalence of NAFLD and liver fibrosis among PLWH. Other information was also extracted from the articles, including the following: author, year of publication, type of study, study location, sample size, mean age of the participants, sex distribution, percentage of participants who smoked, mean body mass index (BMI), mean waist circumference, percentage of overweight participants, percentage of participants with metabolic syndrome, homeostasis model assessment-IR (HOMA-IR) levels, percentages of participants with diabetes mellitus, hypertension and dyslipidemia, triglyceride (TG), total cholesterol (TC), high-density lipoprotein (HDL), low-density lipoprotein (LDL), fasting glucose, alanine transaminase (ALT), aspartate transaminase (AST), and γ-glutamyl transpeptidase (GGT) levels, duration of HIV infection, duration of ART, current ART use, undetectable HIV RNA status, current CD4^+^ T-cell count, nadir CD4^+^ T-cell count, and the percentages of participants on nucleoside reverse transcriptase inhibitors (NRTIs), protease inhibitors (PIs), and integrase inhibitors (INIs).

### Statistical analysis

We adopted Comprehensive Meta-Analysis (CMA) Version 3.0 (Biostat, Englewood, New Jersey) to conduct the quantitative analysis. The combined event rate (ER) and random effects meta-analysis were conducted on the basis of the number of individuals with NAFLD, the number of individuals with liver fibrosis, and the total sample size. Egger’s rank correlation test was used to assess publication bias across studies. I^2^ and Q tests were used to assess the proportion and statistical significance of heterogeneity. The threshold for statistical significance was a 2-tailed p value < 0.05.

## Quality Assessment

The Agency of Healthcare Research and Quality (AHRQ) methodology checklist (http://www.ncbi.nlm.nih.Gov/books/NBK35156/) was used to assess study quality. This checklist contains 11 items, and studies are divided into three levels: high-quality (over 8 points), moderate-quality (4–7 points), and low-quality (0–3 points) studies.

### Meta-regression

Our primary outcome was the prevalence of NAFLD and liver fibrosis among PLWH, and potential moderators were the secondary outcomes. The predefined continuous moderators were the mean age of the participants, sex distribution, BMI, waist circumference, diabetes mellitus status, hypertension status, TG, TC, HDL, LDL, fasting glucose, ALT, AST, and GGT levels, duration of ART, current ART use, undetectable HIV RNA status, and proportion of participants on PIs. The categorical moderators were the current CD4^+^ T-cell count and the region where the study was conducted. The restricted maximum likelihood method and Knapp‒Hartung method were used to conduct the meta-regression. We considered *p* < 0.05 to indicate significance.

## Results

### Search results

**Fig. 1:**
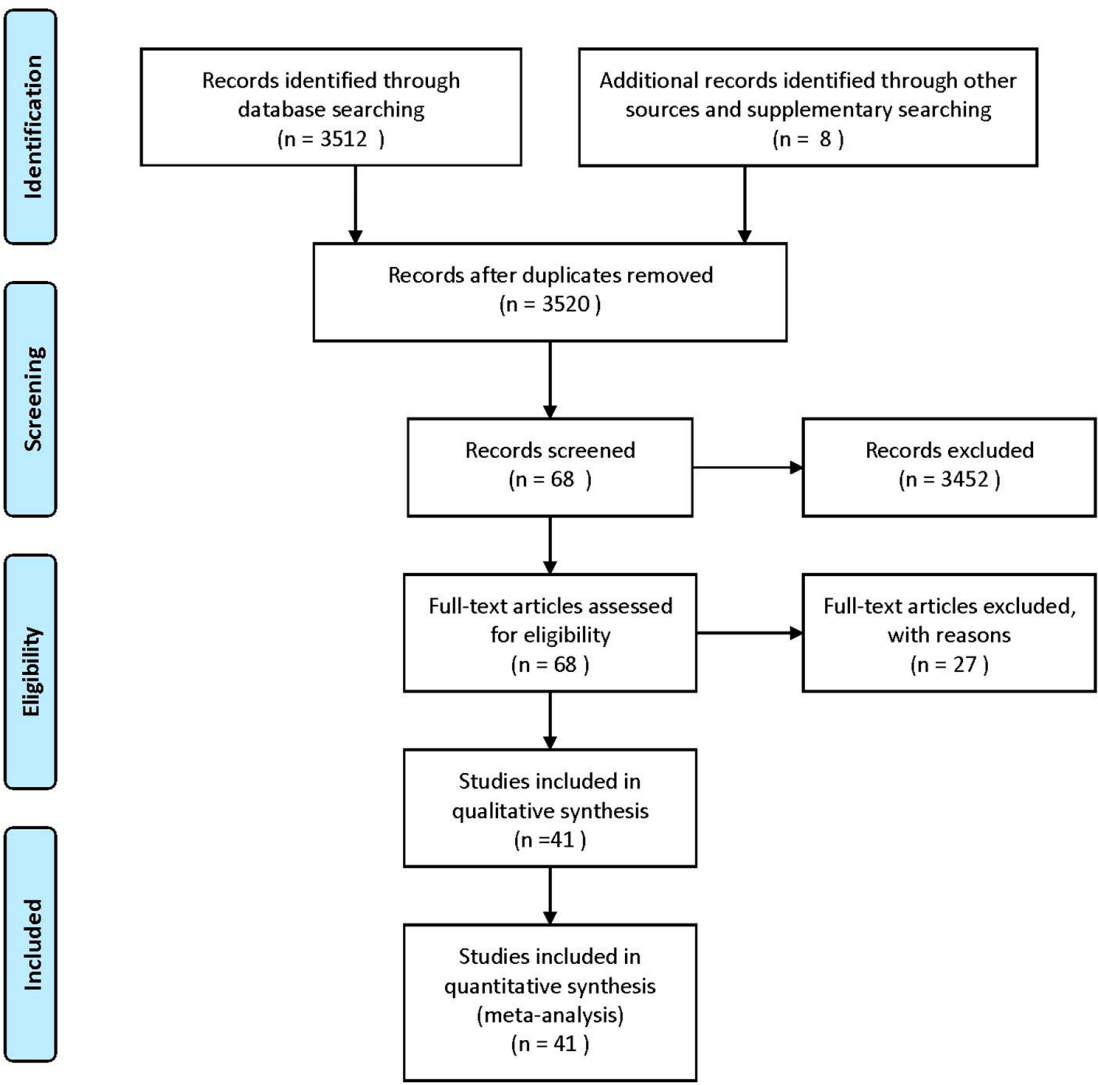
**Flowchart of the study selection process**

This study included a total of 3520 articles. After removing duplicates, 3452 articles were screened on the basis of titles and abstracts. Of these articles, the full texts of 68 studies were assessed. A total of 41 studies [8, 15–54] were included in this meta-analysis. The search strategy is shown in Supplementary Table 2. The reasons for excluded studies are shown in Supplementary Table 3. The flowchart of the study selection process is shown in Fig. 1.

### Study characteristics

A total of 11,026 participants (ranging from 30 [38] to 1511 [17]) whose mean age was 46.7 years (ranging from 27.0 years [47] to 55.0 years [46]) were included in our analysis. The mean proportion of women was 23.0% (ranging from 0.0% [30, 35] to 72.3% [53]). The mean durations of HIV infection and ART were 12.9 years (ranging from 3.0 years [43] to 23.3 years [49]) and 8.6 years (ranging from 1.4 years [27] to 19.0 years [47]), respectively. The mean current CD4^+^ T-cell count and nadir CD4^+^ T-cell count were 584.7 cells/ml (ranging from 325.0 cell/ml [32] to 767.0 cell/ml [52]) and 214.3 cells/ml (ranging from 63.0 cell/ml [52] to 295.1 cell/ml [8]), respectively. The mean percentage of patients with undetectable HIV RNA was 82.8% (ranging from 48.0% [27] to 100.0% [28]). The mean percentage of patients with current ART use was 92.0% (ranging from 65.0% [8] to 100.0% [17, 25, 28, 44, 45, 47, 52]). Among the twenty-three studies, thirty-one [8, 15–17, 19, 21, 22, 24–27, 29–34, 38–43, 45–49, 51, 53, 54] were conducted in a developed country, and ten [18, 20, 23, 28, 35–37, 44, 50, 52] were conducted in a developing country. Thirty-two studies [8, 15–18, 20, 21, 23, 25, 28–32, 36, 38–54] were considered high-quality studies, and nine [19, 22, 24, 26, 27, 33–35, 37] were considered moderate-quality studies. The detailed quality assessment is shown in Supplementary Table 4. The detailed characteristics of the included studies are shown in Table 1.

**Table 1:** Characteristics of the included studies continued form

Study	BMI	Diabetes mellitus, %	Hypertension, %	TG, mg/dl	TC, mg/dl	HDL, mg/dl	LDL, mg/dl	Glucose, mg/dl	ALT, U/L	AST, U/L
Aepfelbacher_2019	27.0	NA	NA	105.0	165.0	50.0	NA	93.0	23.0	25.0
Başaran_2023	26.1	NA	NA	127.0	201.1	43.9	135.0	92.8	19.5	21.5
Benmassaoud_2018	26.8	9.7	21.1	154.9	187.7	47.2	104.1	NA	21.2	27.4
Bischoff_2021	24.9	NA	NA	187.6	197.6	49.4	121.4	95.2	35.4	24.5
Cervo_2020	25.0	14.7	25.2	141.8	181.9	50.3	NA	93.6	25.9	23.6
Crum-Cianflone_2009	26.0	NA	NA	172.1	185.9	40.3	113.8	91.5	NA	NA
De_2022	22.9	6	8	NA	187.6	48.3	NA	102.2	NA	NA
De Almeida_2021	25.0	10.2	22.2	124.0	185.0	43.0	112.0	93.0	29.0	25.0
Fourman_2021	31.0	NA	47.0	142.5	NA	NA	NA	95.7	30.8	32.1
Guaraldi_2008	23.8	NA	13.8	195.2	198.1	45.3	127.7	99.0	NA	NA
Han_2023	22.6	NA	NA	109.0	195.0	45.0	118.0	NA	29.0	25.0
Ingiliz_2008	23.0	NA	NA	194.0	176.9	NA	NA	91.8	80.0	58.0
Jongraksak_2021	22.8	6.7	NA	149.2	202.2	49.8	124.6	95.8	31.4	38.0
Kaplan_2020	29.1	19.0	42.2	154.9	187.7	47.0	107.1	NA	61.0	48.0
Kirkegaard-Klitbo_2020	24.7	7.3	NA	159.5	189.6	NA	108.4	NA	NA	NA
Lallukka-Brück_2020	23.1	NA	NA	168.3	NA	50.3	120	NA	NA	NA
Lemoine_2017	24.6	8.2	NA	167.5	190.4	46.8	114.2	98.5	34.0	29.0
Lemoine_2022	27.0	NA	48.0	141.8	NA	45.3	NA	95.4	34.0	29.0
Liu_2021	22.6	8.0	10.0	143.5	180.7	41.0	NA	NA	29.0	NA
Lombardi_2016	24.6	6.3	9.7	137.0	210.0	42.0	136.0	NA	26.0	23.0
Lombardi_2017		11.0	18.2	NA	NA	NA	NA	NA	56.0	41.0
Lui_2016	23.6	48.8	41.3	159.5	185.8	42.6	100.6	99	NA	NA
Maurice_2020	29.2	21.2	44.9	173.9	178.0	46.4	104.5	106.2	68.0	46.0
Milic_2020	24.6	18.2	NA	137.2	179.5	50.8	116.6	NA		24.7
Mohr_2018	24.0	4.0	NA	132.0	198.0	46.0	119.0	NA	30.0	21.0
Morse_2015	27.6	3.0	NA	191.0	193.0	39.0	NA	NA	72.0	46.0
Nishijima_2014	22.1	5.0	20.0	162.0	175.0	44.0	102.0	NA	26.0	25.0
Pezzini_2021	25.7	35.7	27.6	164.1	188.0	44.7	103.0	NA	21.0	23.0
Prat_2019	27.0	11.0	21.0	150.6	189.6	50.3	91.8	NA	NA	NA
Price_2019	26.0	12.0	48.0	130.0	NA	NA	NA	NA	25.0	24.0
Price_2017	26.0	11.0	49.0	131.0	NA	46.0	107.0	NA	25.0	23.0
Riebensahm_2022	25.9	13.2	34.0	174.7	NA	NA	NA	NA	30.7	NA
Sebastiani_2022	29.9	26.7	36.7	186.5	167.9	45.4	90.5	104.2	40.8	30.0
Shur_2016	26.7	12.2	NA	256.9	209.0	46.4	116.1	100.8	NA	NA
Sim_2021	27.9	NA	NA	96.0	183.6	52.1	105.0	89.6	25.0	26.0
Villa_2021	23.9	NA	NA	106.3	181.9	50.3	104.5	NA	18.0	27.0
Vodkin_2015	29.8	18.2	48.5	242.1	207.5	39.4	119.6	100.7	146.2	88.4
Vuille-Lessard_2016	25.7	11.3	16.3	127.6	185.8	44.5	104.5	95.4	28.9	25.5
Vujanovic_2019	24.8	NA	NA	NA	126.9	44.5	130.0	92.2	30.5	26.2
Yanavich_2021	26.1	NA	NA	124.0	NA	NA	NA	94.0	NA	NA
Zizza_2017	NA	5.4	17.9	NA	NA	NA	NA	NA	NA	NA

TE: Transient elastography; MRI-PDFF: MRI-derived proton density fat fraction; MRE: Magnetic resonance elastography; US: Ultrasound; LB: Liver biopsy; CT: Computed tomography; H-MRS: Magnetic resonance spectroscopy; NA: Not available

### NAFLD prevalence

**Fig. 2:**
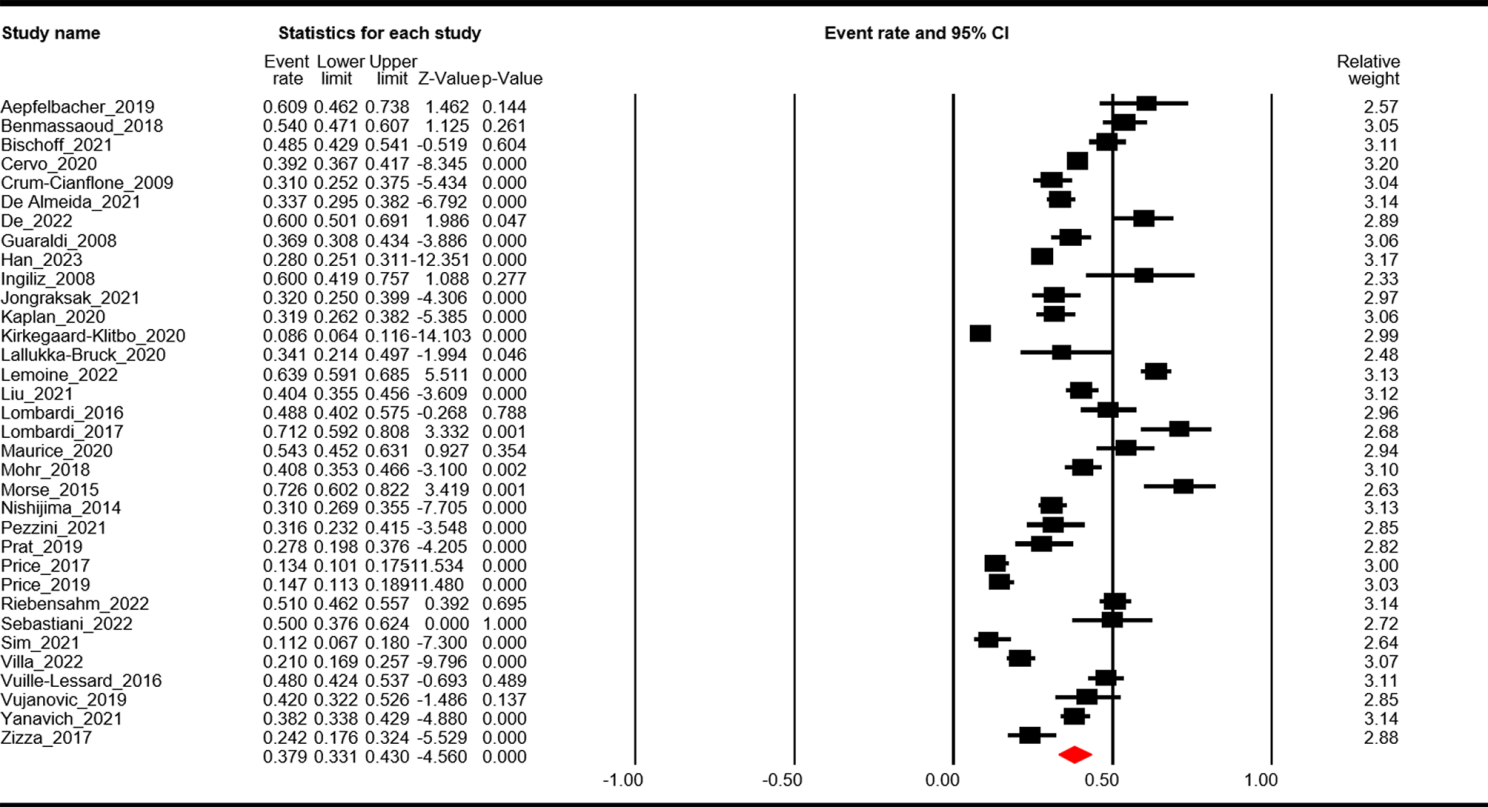
Pooled prevalence of NAFLD

Thirty-four studies [8, 15–21, 23–31, 33–39, 42–48, 50, 51, 53, 54] reported the prevalence of NAFLD, of which the combined ER was 0.38 (95% CI: 0.33–0.43, *p* < 0.01). Significant heterogeneity was found (Q = 703.3, I^2^ = 95.3, *p* < 0.01). Egger’s regression test revealed no publication bias (intercept=-0.07, *p* = 0.97). See Fig. 2 and Supplementary Fig. 1.

### Factors associated with NAFLD

A significantly elevated TG level was associated with NAFLD (SE = 0, *p* = 0.04; *n* = 30). The other factors showed no significant associations. See Supplementary Table 5.

### Prevalence of liver fibrosis

**Fig. 3:**
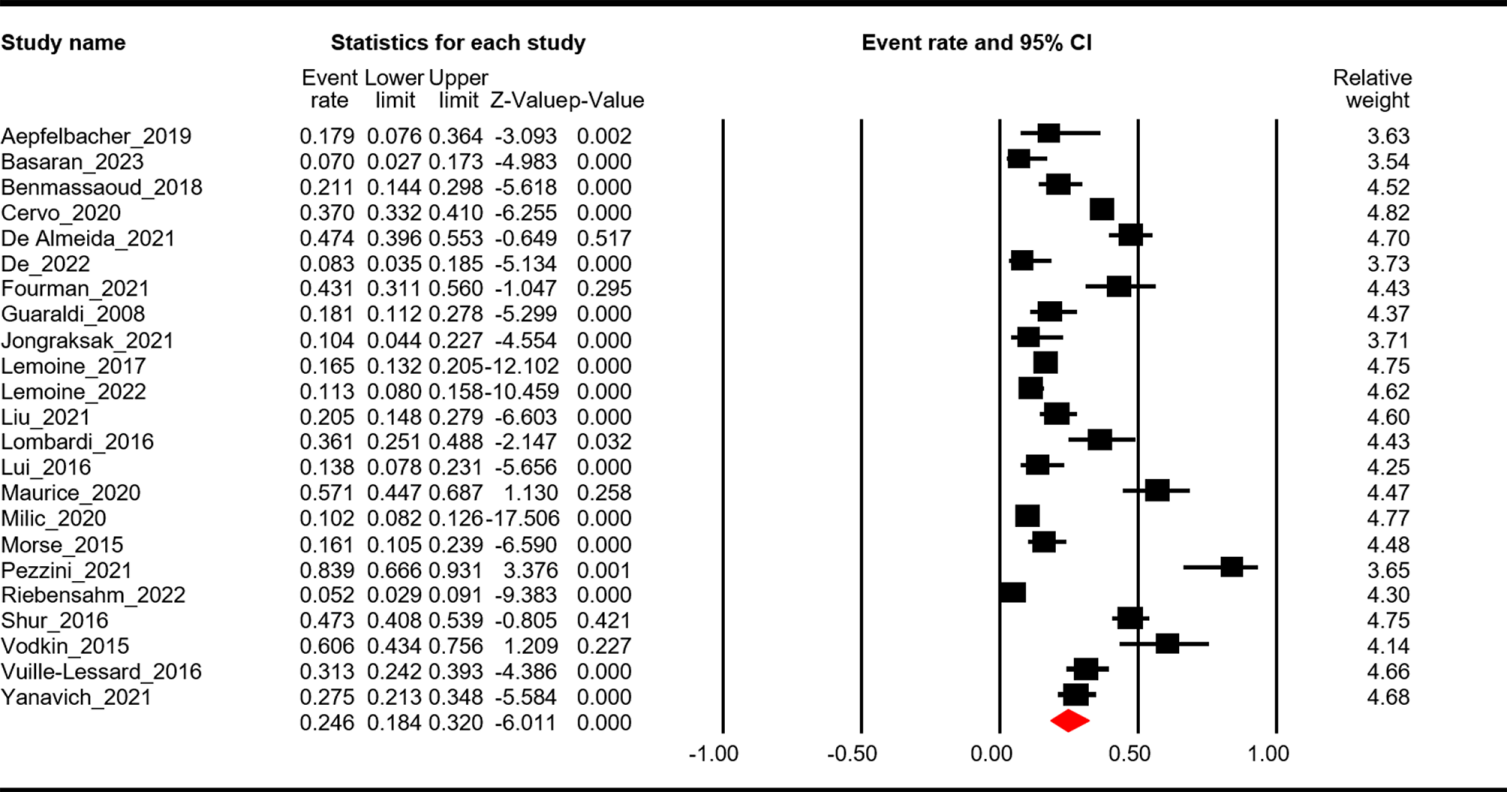
Pooled prevalence of liver fibrosis

Twenty-three studies [15, 17–20, 22–25, 28, 32, 34, 36, 39–43, 46, 47, 49, 50, 52] reported the prevalence of liver fibrosis, of which the combined ER was 0.25 (95% CI: 0.18–0.32, *p* < 0.01). Significant heterogeneity was found (Q = 411.6, I^2^ = 94.7, *p* < 0.01). Egger’s regression test revealed no publication bias (intercept=-1.4, *p* = 0.48). See Fig. 3 and Supplementary Fig. 2.

### Factors associated with liver fibrosis

The results of the subgroup analysis revealed that the prevalence of liver fibrosis varied among patients diagnosed with different diagnostic methods (Q = 55.6 l, *p* < 0.01). See Supplementary Table 5.

## Discussion

Using meta-analysis, we synthesized evidence from a total of 41 studies to comprehensively and systematically evaluate the prevalence and disease progression of NAFLD in the HIV population worldwide. We found that in PLWH, the prevalence of NAFLD ranged from 33 to 43%, a high triglyceride level was the main risk factor, and the prevalence of fibrosis in the NAFLD population ranged from 18 to 32%. Our data indicate that more than one-third of individuals with HIV have fatty liver, which should be given sufficient attention.

The results of the subgroup analysis revealed that liver biopsy might be the best way to confirm liver fibrosis, with a prevalence ranging from 48 to 68%. Liver biopsy can directly reveal pathological changes in liver tissue and be used to make a more accurate diagnosis, but it is an invasive examination that has stricter use requirements than imaging examinations do. Therefore, the subjects who underwent liver biopsy were mostly those who had symptoms related to liver disease or abnormalities that could not be confirmed by imaging examinations. These findings explain the high incidence of liver fibrosis diagnosed with this diagnostic method. In the absence of severe conditions, patients with NAFLD generally remain asymptomatic, obviating the need for routine liver biopsy. Moreover, imaging findings and liver biopsy demonstrate high diagnostic accuracy for NAFLD the diagnostic accuracy of imaging findings and liver biopsy. Thus, there is no disparity in the prevalence of NAFLD between patients diagnosed by these two methods.

In our study, the prevalence of NAFLD in PLWH in Europe was the highest, at approximately 54.3%, and the data were obtained from patients with abnormal liver function who further underwent liver biopsy due to clinical suspicion of liver fibrosis [25]. Canada had the second highest prevalence (54%), with data from patients diagnosed by TE with the controlled attenuation parameter (CAP), a widely used noninvasive technique to quantify liver fat [55, 56]. Sim and Price reported that the United States had the lowest prevalence rates (11.2% and 13.4%, respectively). Both studies used CT scan as the diagnostic method [31, 33], which is rarely used for the detection of hepatic steatosis because it has limited sensitivity for mild hepatic steatosis (liver fat content less than 30%) and exposes patients to radiation [57]. At present, MRI-PDFF or H-MRS are more accurate than liver biopsy in the diagnosis of hepatic status, but they are currently limited in terms of their use in clinical studies and have not been commercially promoted [58, 59].

In HIV-negative populations, many studies have shown that BMI, age, and metabolic syndrome are associated with an increased risk of hepatic steatosis [34, 60, 61]. However, few studies have reported on predictors of steatosis and its risk factors in PLWH [62, 63]. Previous meta-analyses have shown that high BMI, a high waist circumference, type 2 diabetes, hypertension, a high triglyceride level and a high CD4^+^ T-cell count are associated with NAFLD, whereas the HIV viral load, duration of HIV infection and ART status are not associated with NAFLD [64]. The results of the regression analysis in our study suggested that triglyceride levels were associated with fatty liver in PLWH, which may indicate that metabolic disorders are among the predictive factors for the occurrence and development of HIV-related NAFLD. Recent studies have reported that the prevalence of dyslipidemia in the PLWH cohort was greater than that in the general population [65, 66]. Triglycerides are deposited in the liver directly and can also promote the occurrence of fatty liver by affecting the insulin sensitivity of fatty and muscle tissue. Previous WHO guidelines recommended that ART regimens be based mainly on the nonnucleoside reverse transcriptase inhibitor Efavirenz, with the main adverse effect being elevated triglyceride levels. The incidence of NAFLD gradually increases with age, which may cause more serious health risks in PWLH owing to their relatively young ages [67]. Therefore, the triglyceride level is recommended as a routine monitoring index for PLWH.

One of the very interesting findings of our study was that the indicators associated with central obesity, such as waist circumference and BMI, were not significantly different across studies, suggesting that PLWH may mainly have lean fatty liver, which is consistent with the results of previous studies. Studies reported that the prevalence of lean NAFLD in PLWH was 13.9% [17, 68], and the average BMI of these NAFLD patients was lower than that of HIV-negative NAFLD patients. A histological comparison revealed that the severity of NAFLD in PLWH was greater than that in HIV-negative NAFLD patients [40]. Although the BMI of PLWH receiving ART did not exceed the threshold for obesity, steatosis could still occur, especially in males with a body mass index greater than 23.0 kg/m^2^ [16]. Therefore, further large-scale studies are needed to clarify the BMI risk threshold that affects the occurrence of fatty liver disease in PLWH.

PLWH have unique risk factors for NAFLD, including HIV infection itself, inflammation, and ART side effects. Owing to the persistent presence of the HIV reservoir, the virus continues to activate the immune system in HIV-positive individuals, resulting in inflammation that leads to disorders of lipid metabolism, which in turn leads to fatty liver disease. Adverse reactions to early ART drugs can cause mitochondrial toxicity, resulting in lipodystrophy secondary to hypertriglyceridemia, IR, hepatic mitochondrial toxicity, lactate elevation, and liver fat changes [69, 70]. Studies from the era of INSTIs have shown that the nucleoside analog tenofovir is associated with weight loss in patients, whereas INIs can lead to weight gain. However, a recent study revealed that weight gain caused by INIs and tenofovir alafenamide (TAF) was associated with the development of fatty liver [16]. However, the conclusions of other studies are controversial. The risk of coexisting metabolic and cardiovascular diseases has gradually increased for patients receiving ART treatment for decades. This study did not find a correlation between fatty liver disease and ART, which may be closely related to individual dietary habits.

The severity of liver fibrosis is the strongest predictor of disease-specific mortality in HIV-negative NAFLD patients, and cardiovascular disease is one of the main causes of death in this population [71]. However, research on the prevalence and risk factors for liver fibrosis in HIV-positive NAFLD patients is limited. The risk of liver fibrosis in PLWH has been reported to be twice that in HIV-negative individuals [34, 72]. In this meta-analysis, we found that the prevalence of liver fibrosis in HIV-positive NAFLD patients was 24.6%, which was relatively high. Therefore, regular monitoring of patients with liver fibrosis may be an effective measure to prevent the progression of NAFLD in PLWH.

Studies on HIV-negative people have shown that a high HOMA-IR value is another important risk factor for the development of NAFLD. Numerous studies have suggested that NAFLD patients with IR have an increased risk of developing NASH and fibrosis, leading to increased all-cause and liver-related mortality [73]. In addition, IR is an important pathogenic mechanism involved in the occurrence and progression of fatty liver. The ViiV Healthcare trial evaluated the change in the HOMA-IR values of PLWH and reported that 80% of the population had a base HOMA-IR value above 2.0, which further increased during the follow-up period. Most studies reported that high HOMA-IR values may be related to ART [74–76]. Compared with that in non-HIV-infected individuals, the change in the HOMA-IR value was more obvious in PLWH. Therefore, IR was included as a risk factor in this meta-analysis, but owing to the limited number of relevant studies, no positive results were obtained, so more clinical studies are needed for further verification in the future.

This meta-analysis also has several limitations. The lack of adequate original research led to low statistical power in the subgroup analysis. Additionally, this meta-analysis aimed to clarify the association between NAFLD and INI use. Given the increasing use of INIs, this may be a significant limitation. We also failed to compare the difference in NAFLD incidence between PLWH and HIV-negative people, which might lead to ignorance of the role of HIV in the progression of NAFLD. Finally, there is also a lack of relevant original research in underdeveloped countries. Most of these countries are in high-AIDS epidemic areas, and the lack of relevant research in these areas makes this analysis less accurate and representative.

## Conclusion

In this meta-analysis, we found that the prevalence rates of NAFLD and liver fibrosis in PLWH were greater than those in the general population. This means that PLWH is more likely to suffer from cirrhosis, liver cancer, and extrahepatic complications, which require sufficient attention. In addition, noninvasive imaging monitoring of NAFLD changes should be strengthened, and a high TG level might be an early predictive indicator for HIV-associated fatty liver disease; however, large-scale prospective clinical research data are still needed for further validation and evaluation.

## Electronic supplementary material

Below is the link to the electronic supplementary material.

Supplementary Material 1

Supplementary Material 2

## Data Availability

Raw data is provided within the supplementary information files, naming “NAFLD raw data. xlsx”.

## Abbreviations

AIDS: Acquired immune deficiency syndrome
HIV: Human immunodeficiency virus
ART: Antiretroviral therapy
CVD: Cardiovascular disease
CLD: Chronic liver disease
D: A:D: Data collection on Adverse events of Anti-HIV Drugs
NAFLD: Non-alcoholic fatty liver disease
T2DM: Diabetes mellitus type 2
MetS: Metabolic syndrome
IR: Insulin resistance
PRISMA: Preferred Reporting Items for Systematic Reviews and Meta-Analysis
BMI: Body mass index
HOMA-IR: Homeostasis model assessment-IR
TG: Triglyceride
TC: Total cholesterol
HDL: High-density lipoprotein
LDL: Low-density lipoprotein
ALT: Alanine transaminase
AST: Aspartate transaminase
GGT: γ-glutamyl transpeptidase
NRTI: Nucleoside reverse transcriptase inhibitors
PI: Protease inhibitor
INIS: Integrase inhibitors
CMA: Comprehensive Meta-Analysis
ER: Event rate
AHRQ: The Agency of Healthcare Research and Quality
TAF: Tenofovir alafenamide
