# Supplementary material for: The prevalence of nonalcoholic fatty liver disease in people living with HIV: a systematic review and meta-analysis
Source: BMC Infect Dis. 2025 Feb 19;25:239. doi: 10.1186/s12879-025-10455-y (PMC11921747; doi:10.1186/s12879-025-10455-y)
Supplement: Supplementary file 2 — Supplementary Material 2 [file 12879_2025_10455_MOESM2_ESM.docx]

**Contents**

[**Table 1 PRISMA checklist 2**](#_Toc41334687)

[**Table 2 Full Search Strategy 6**](#_Toc41334688)

[**Table 3 Reasons for Excluding in Full-text Assessment 7**](#_Toc41334689)

[**Table 4 Study Quality and Risk-of-bias for Included Studies 12**](#_Toc41334691)

[**Table 5 Full Results of Subgroup Analyses 15**](#_Toc41334692)

**Figure 1 Public bias of NAFLD prevalence 24**

**Figure 2 Public bias of liver fibrosis prevalence 25**

**Table 1 PRISMA checklist**

| **Section/topic** | | **#** | | **Checklist item** | | **Reported on page #** |
| --- | --- | --- | --- | --- | --- | --- |
| **TITLE** | | | | | |  |
| Title | | 1 | | Identify the report as a systematic review, meta-analysis, or both. | | Page 1 Title Page |
| **ABSTRACT** | | | | | |  |
| Structured summary | | 2 | | Provide a structured summary including, as applicable: background; objectives; data sources; study eligibility criteria, participants, and interventions; study appraisal and synthesis methods; results; limitations; conclusions and implications of key findings; systematic review registration number. | | Page 2 and 3 Abstract Page |
| **INTRODUCTION** | | | | | |  |
| Rationale | | 3 | | Describe the rationale for the review in the context of what is already known. | | Page 4-5 |
| Objectives | | 4 | | Provide an explicit statement of questions being addressed with reference to participants, interventions, comparisons, outcomes, and study design (PICOS). | | Page 5 |
| **METHODS** | | | | | |  |
| Protocol and registration | | 5 | | Indicate if a review protocol exists, if and where it can be accessed (e.g., Web address), and, if available, provide registration information including registration number. | | Page 5 |
| Eligibility criteria | | 6 | | Specify study characteristics (e.g., PICOS, length of follow-up) and report characteristics (e.g., years considered, language, publication status) used as criteria for eligibility, giving rationale. | | Page 6 |
| Information sources | | 7 | | Describe all information sources (e.g., databases with dates of coverage, contact with study authors to identify additional studies) in the search and date last searched. | | Page 6 and Supplementary Table 2 |
| Search | | 8 | | Present full electronic search strategy for at least one database, including any limits used, such that it could be repeated. | | Page 6 and Supplementary Table 2 |
| Study selection | | 9 | | State the process for selecting studies (i.e., screening, eligibility, included in systematic review, and, if applicable, included in the meta-analysis). | | Page 6, Figure 1 and Supplementary Table 3 |
| Data collection process | | 10 | | Describe method of data extraction from reports (e.g., piloted forms, independently, in duplicate) and any processes for obtaining and confirming data from investigators. | | Page 6 |
| Data items | | 11 | | List and define all variables for which data were sought (e.g., PICOS, funding sources) and any assumptions and simplifications made. | | Page 6-7 |
| Risk of bias in individual studies | | 12 | | Describe methods used for assessing risk of bias of individual studies (including specification of whether this was done at the study or outcome level), and how this information is to be used in any data synthesis. | | Page 7 and Supplementary Table 5 |
| Summary measures | | 13 | | State the principal summary measures (e.g., risk ratio, difference in means). | | Page 7 |
| Synthesis of results | | 14 | | Describe the methods of handling data and combining results of studies, if done, including measures of consistency (e.g., I^2^) for each meta-analysis. | | Page 7 |
| Section/topic | | # | | Checklist item | | Reported on page # |
| Risk of bias across studies | | 15 | | Specify any assessment of risk of bias that may affect the cumulative evidence (e.g., publication bias, selective reporting within studies). | | Page 7 |
| Additional analyses | | 16 | | Describe methods of additional analyses (e.g., sensitivity or subgroup analyses, meta-regression), if done, indicating which were pre-specified. | | Page 7-8 |
| **RESULTS** | | | | |  | |
| Study selection | 17 | | Give numbers of studies screened, assessed for eligibility, and included in the review, with reasons for exclusions at each stage, ideally with a flow diagram. | | Page 8 and Figure 1 | |
| Study characteristics | 18 | | For each study, present characteristics for which data were extracted (e.g., study size, PICOS, follow-up period) and provide the citations. | | Page 8 and Table 1 | |
| Risk of bias within studies | 19 | | Present data on risk of bias of each study and, if available, any outcome level assessment (see item 12). | | Page 9 and Supplementary Figure 1&2 | |
| Results of individual studies | 20 | | For all outcomes considered (benefits or harms), present, for each study: (a) simple summary data for each intervention group (b) effect estimates and confidence intervals, ideally with a forest plot. | | Page 9 and Figure 2&3 | |
| Synthesis of results | 21 | | Present results of each meta-analysis done, including confidence intervals and measures of consistency. | | Page 9 and Figure 2&3 | |
| Risk of bias across studies | 22 | | Present results of any assessment of risk of bias across studies (see Item 15). | | Page 9 | |
| Additional analysis | 23 | | Give results of additional analyses, if done (e.g., sensitivity or subgroup analyses, meta-regression [see Item 16]). | | Page 9 and Supplementary Table 5 | |
| **DISCUSSION** | | | | |  | |
| Summary of evidence | 24 | | Summarize the main findings including the strength of evidence for each main outcome; consider their relevance to key groups (e.g., healthcare providers, users, and policy makers). | | Page 9-13 | |
| Limitations | 25 | | Discuss limitations at study and outcome level (e.g., risk of bias), and at review-level (e.g., incomplete retrieval of identified research, reporting bias). | | Page 13 | |
| Conclusions | 26 | | Provide a general interpretation of the results in the context of other evidence, and implications for future research. | | Page 13-14 | |
| **FUNDING** | | | | |  | |
| Funding | 27 | | Describe sources of funding for the systematic review and other support (e.g., supply of data); role of funders for the systematic review. | | Page 14 | |

*From:*  Moher D, Liberati A, Tetzlaff J, Altman DG, The PRISMA Group (2009). Preferred Reporting Items for Systematic Reviews and Meta-Analyses: The PRISMA Statement. PLoS Med 6(7): e1000097. doi:10.1371/journal.pmed1000097

For more information, visit: **www.prisma-statement.org**.

**Table 2 Full Search Strategy**

| **Database** | **Time** | **Strategy** | **Outcome** |
| --- | --- | --- | --- |
| Web of science | 2023.8.15 | "human immune deficiency virus" OR "Acquired immune deficiency syndrome" OR "HIV" OR "AIDS"(Abstract) and "fatty liver" OR "non alcoholic liver" OR "nafld" OR "nail" OR "NASH" OR "steatohepatitis"( Abstract) | 1032 |
| Pubmed | 2023.8.15 | ("human immune deficiency virus" OR "Acquired immune deficiency syndrome" OR "HIV" OR "AIDS") AND ("fatty liver" OR "non alcoholic liver" OR "nafld" OR "nail" OR "NASH" OR "steatohepatitis") | 1332 |
| Embase | 2023.8.15 | ('human immune deficiency virus':ab,ti OR 'acquired immune deficiency syndrome':ab,ti OR 'hiv':ab,ti OR 'aids':ab,ti) AND ('non alcoholic liver':ab,ti OR 'nafld':ab,ti OR 'nail':ab,ti OR 'nash':ab,ti OR 'steatohepatitis':ab,ti) | 963 |
| Cochrane Library | 2023.8.15 | "human immune deficiency virus" OR "Acquired immune deficiency syndrome" OR "HIV" OR "AIDS" in Title Abstract Keyword AND "fatty liver" OR "non alcoholic liver" OR "nafld" OR "nail" OR "NASH" OR "steatohepatitis" in Title Abstract Keyword | 185 |

**Table 3 Reasons for Excluding in Full-text Assessment**

| No. | Full text being excluded | Reasons |
| --- | --- | --- |
| 1 | Androutsakos T, Schina M, Pouliakis A, Kontos A, Sipsas N, Hatzis G. Liver Fibrosis Assessment in a Cohort of Greek HIV Mono-Infected Patients by Non-Invasive Biomarkers. Curr HIV Res. 2019;17(3):173-182. | Being a method evaluation |
| 2 | Ajmera VH, Cachay ER, Ramers CB, Bassirian S, Singh S, Bettencourt R, Richards L, Hamilton G, Middleton M, Fowler K, Sirlin C, Loomba R. Optimal Threshold of Controlled Attenuation Parameter for Detection of HIV-Associated NAFLD With Magnetic Resonance Imaging as the Reference Standard. Clin Infect Dis. 2021 Jun 15;72(12):2124-2131. | Being a method evaluation |
| 3 | Arendt BM, Mohammed SS, Ma DW, Aghdassi E, Salit IE, Wong DK, Guindi M, Sherman M, Heathcote EJ, Allard JP. Non-alcoholic fatty liver disease in HIV infection associated with altered hepatic fatty acid composition. Curr HIV Res. 2011 Mar;9(2):128-35. | Not having extracted data |
| 4 | Busca C, Sánchez-Conde M, Rico M, Rosas M, Valencia E, Moreno A, Moreno V, Martín-Carbonero L, Moreno S, Pérez-Valero I, Bernardino JI, Arribas JR, González J, Olveira A, Castillo P, Abadía M, Guerra L, Mendez C, Montes ML. Assessment of Noninvasive Markers of Steatosis and Liver Fibrosis in Human Immunodeficiency Virus-Monoinfected Patients on Stable Antiretroviral Regimens. Open Forum Infect Dis. 2022 Jun 9;9(7):ofac279. | Being a method evaluation |
| 5 | Calza L, Colangeli V, Borderi M, Coladonato S, Tazza B, Fornaro G, Badia L, Guardigni V, Verucchi G, Viale P. Improvement in liver steatosis after the switch from a ritonavir-boosted protease inhibitor to raltegravir in HIV-infected patients with non-alcoholic fatty liver disease. Infect Dis (Lond). 2019 Aug;51(8):593-601. | Not having extracted data |
| 6 | Fernandez-Fuertes M, Macías J, Corma-Gómez A, Rincón P, Merchante N, Gómez-Mateos J, Pineda JA, Real LM. Similar prevalence of hepatic steatosis among patients with chronic hepatitis C with and without HIV coinfection. Sci Rep. 2020 Apr 21;10(1):6736. | Not having extracted data |
| 7 | Fernandez-Botran R, Plankey MW, Ware D, Bordon J. Changes in liver steatosis in HIV-positive women are associated with the BMI, but not with biomarkers. Cytokine. 2021 Aug;144:155573. | Not having extracted data |
| 8 | Gabriel CL, Ye F, Fan R, Nair S, Terry JG, Carr JJ, Silver H, Baker P, Hannah L, Wanjalla C, Mashayekhi M, Bailin S, Lima M, Woodward B, Izzy M, Ferguson JF, Koethe JR. Hepatic Steatosis and Ectopic Fat Are Associated With Differences in Subcutaneous Adipose Tissue Gene Expression in People With HIV. Hepatol Commun. 2021 Feb 27;5(7):1224-1237. | Not having extracted data |
| 9 | Gawrieh S, Corey KE, Lake JE, Samala N, Desai AP, Debroy P, Sjoquist JA, Robison M, Tann M, Akisik F, Bhamidipalli SS, Saha CK, Zachary K, Robbins GK, Gupta SK, Chung RT, Chalasani N. Non-alcoholic fatty liver disease is not associated with impairment in health-related quality of life in virally suppressed persons with human immune deficiency virus. PLoS One. 2023 Feb 10;18(2):e0279685. | Not having extracted data |
| 10 | Guaraldi G, Lonardo A, Ballestri S, Zona S, Stentarelli C, Orlando G, Carli F, Carulli L, Roverato A, Loria P. Human immunodeficiency virus is the major determinant of steatosis and hepatitis C virus of insulin resistance in virus-associated fatty liver disease. Arch Med Res. 2011 Nov;42(8):690-7. | Not having extracted data |
| 11 | Kaplan A, Simon TG, Henson JB, Wang T, Zheng H, Osganian SA, Rosenblatt R, Lake J, Corey KE. Brief Report: Relationship Between Nonalcoholic Fatty Liver Disease and Cardiovascular Disease in Persons With HIV. J Acquir Immune Defic Syndr. 2020 Aug 1;84(4):400-404. | Being a brief report |
| 12 | Khalili M, King WC, Kleiner DE, Jain MK, Chung RT, Sulkowski M, Lisker-Melman M, Wong DK, Ghany M, Sanyal A, Sterling RK. Fatty Liver Disease in a Prospective North American Cohort of Adults With Human Immunodeficiency Virus and Hepatitis B Virus Coinfection. Clin Infect Dis. 2021 Nov 2;73(9):e3275-e3285. | Having the same dataset published in previous studies |
| 13 | Krahn T, Martel M, Sapir-Pichhadze R, Kronfli N, Falutz J, Guaraldi G, Lebouche B, Klein MB, Wong P, Deschenes M, Ghali P, Sebastiani G. Nonalcoholic Fatty Liver Disease and the Development of Metabolic Comorbid Conditions in Patients With Human Immunodeficiency Virus Infection. J Infect Dis. 2020 Aug 4;222(5):787-797. | Having the same dataset published in previous studies |
| 14 | Krishnan A, Sims OT, Surapaneni PK, Woreta TA, Alqahtani SA. Risk of Adverse cardiovascular outcomes among persons living with HIV and nonalcoholic fatty liver disease: a multicenter matched cohort study. AIDS. 2023 Mar 6. | Having the same dataset published in previous studies |
| 15 | Lemoine M, Barbu V, Girard PM, Kim M, Bastard JP, Wendum D, Paye F, Housset C, Capeau J, Serfaty L. Altered hepatic expression of SREBP-1 and PPARgamma is associated with liver injury in insulin-resistant lipodystrophic HIV-infected patients. AIDS. 2006 Feb 14;20(3):387-95. | Not having extracted data |
| 16 | Lemoine M, Assoumou L, De Wit S, Girard PM, Valantin MA, Katlama C, Necsoi C, Campa P, Huefner AD, Schulze Zur Wiesch J, Rougier H, Bastard JP, Stocker H, Mauss S, Serfaty L, Ratziu V, Menu Y, Schlue J, Behrens G, Bedossa P, Capeau J, Ingiliz P, Costagliola D; ANRS-ECHAM Group. Diagnostic Accuracy of Noninvasive Markers of Steatosis, NASH, and Liver Fibrosis in HIV-Monoinfected Individuals at Risk of Nonalcoholic Fatty Liver Disease (NAFLD): Results From the ECHAM Study. J Acquir Immune Defic Syndr. 2019 Apr 1;80(4):e86-e94. | Having the same dataset published in previous studies |
| 17 | Macías J, Real LM, Rivero-Juárez A, Merchante N, Camacho A, Neukam K, Rivero A, Mancebo M, Pineda JA. Changes in liver steatosis evaluated by transient elastography with the controlled attenuation parameter in HIV-infected patients. HIV Med. 2016 Nov;17(10):766-773. | Not having extracted data |
| 18 | Moreno-Perez O, Reyes-Garcia R, Muñoz-Torres M, Merino E, Boix V, Reus S, Giner L, Alfayate R, Garcia-Fontana B, Sanchez-Paya J, Picó A, Portilla J. High Irisin levels in nondiabetic HIV-infected males are associated with insulin resistance, nonalcoholic fatty liver disease, and subclinical atherosclerosis. Clin Endocrinol (Oxf). 2018 Oct;89(4):414-423. | Not having extracted data |
| 19 | Navarro J, Curran A, Raventós B, García J, Suanzes P, Descalzo V, Álvarez P, Espinosa N, Montes ML, Suárez-García I, Amador C, Muga R, Falcó V, Burgos J; Spanish HIV Research Network (CoRIS). Prevalence of non-alcoholic fatty liver disease in a multicentre cohort of people living with HIV in Spain. Eur J Intern Med. 2023 Apr;110:54-61. | Not having extracted data |
| 20 | Pembroke T, Deschenes M, Lebouché B, Benmassaoud A, Sewitch M, Ghali P, Wong P, Halme A, Vuille-Lessard E, Pexos C, Klein MB, Sebastiani G. Hepatic steatosis progresses faster in HIV mono-infected than HIV/HCV co-infected patients and is associated with liver fibrosis. J Hepatol. 2017 Oct;67(4):801-808. | Not having extracted data |
| 21 | Perazzo H, Cardoso SW, Yanavich C, Nunes EP, Morata M, Gorni N, da Silva PS, Cardoso C, Almeida C, Luz P, Veloso VG, Grinsztejn B. Predictive factors associated with liver fibrosis and steatosis by transient elastography in patients with HIV mono-infection under long-term combined antiretroviral therapy. J Int AIDS Soc. 2018 Nov;21(11):e25201. | Not having extracted data |
| 22 | Sebastiani G, Cocciolillo S, Mazzola G, Malagoli A, Falutz J, Cervo A, Petta S, Pembroke T, Ghali P, Besutti G, Franconi I, Milic J, Cascio A, Guaraldi G. Application of guidelines for the management of nonalcoholic fatty liver disease in three prospective cohorts of HIV-monoinfected patients. HIV Med. 2020 Feb;21(2):96-108. | Having the same dataset published in previous studies |
| 23 | Schwarz C, Chromy D, Bauer D, Duong N, Schmidbauer VU, Schwarz M, Mandorfer M, Rieger A, Trauner M, Gschwantler M, Reiberger T. Prevalence and dynamics of NAFLD-associated fibrosis in people living with HIV in Vienna from first presentation to last follow-up. Wien Klin Wochenschr. 2023 Aug;135(15-16):420-428. | Not having extracted data |
| 24 | Sterling RK, Smith PG, Brunt EM. Hepatic steatosis in human immunodeficiency virus: a prospective study in patients without viral hepatitis, diabetes, or alcohol abuse. J Clin Gastroenterol. 2013 Feb;47(2):182-7. | Not having extracted data |
| 25 | Sudjaritruk T, Bunupuradah T, Aurpibul L, Kosalaraksa P, Kurniati N, Sophonphan J, Trinavarat P, Visrutaratna P, Srinakarin J, Chaijitraruch N, Puthanakit T; NAFLD Study Group. Nonalcoholic fatty liver disease and hepatic fibrosis among perinatally HIV-monoinfected Asian adolescents receiving antiretroviral therapy. PLoS One. 2019 Dec 19;14(12):e0226375. | Not having extracted data |
| 26 | Tamargo JA, Sherman KE, Campa A, Martinez SS, Li T, Hernandez J, Teeman C, Mandler RN, Chen J, Ehman RL, Baum MK. Food insecurity is associated with magnetic resonance-determined nonalcoholic fatty liver and liver fibrosis in low-income, middle-aged adults with and without HIV. Am J Clin Nutr. 2021 Mar 11;113(3):593-601. | Not having extracted data |
| 27 | Torgersen J, So-Armah K, Freiberg MS, Goetz MB, Budoff MJ, Lim JK, Taddei T, Butt AA, Rodriguez-Barradas MC, Justice AC, Kostman JR, Lo Re V 3rd. Comparison of the prevalence, severity, and risk factors for hepatic steatosis in HIV-infected and uninfected people. BMC Gastroenterol. 2019 Apr 15;19(1):52. | Not having extracted data |

**Table 4 Study Quality and Risk-of-bias for Included Studies**

| Study Name | Information Source | Study Criteria | Study Period | Sampling | Interview Method | Instrument Validation | Exclusion Criteria | The Measurement of Confounding Effects | The Process of Dealing with Missing Values | Response Rate | The Use of Follow-up Assessment | Total |
| --- | --- | --- | --- | --- | --- | --- | --- | --- | --- | --- | --- | --- |
| Aepfelbacher_2019 | 1 | 1 | 1 | 1 | 1 | 1 | 1 | 0 | 1 | 1 | 0 | 9 |
| Başaran_2023 | 1 | 1 | 1 | 1 | 1 | 1 | 1 | 0 | 0 | 1 | 1 | 9 |
| Benmassaoud_2018 | 1 | 1 | 1 | 1 | 0 | 1 | 1 | 0 | 1 | 1 | 0 | 8 |
| Bischoff_2021 | 1 | 1 | 1 | 1 | 0 | 1 | 1 | 0 | 0 | 1 | 1 | 8 |
| Cervo_2020 | 1 | 1 | 1 | 1 | 0 | 1 | 1 | 0 | 1 | 1 | 1 | 9 |
| Crum-Cianflone_2009 | 1 | 1 | 1 | 1 | 1 | 1 | 1 | 0 | 1 | 1 | 0 | 9 |
| De_2022 | 1 | 1 | 0 | 1 | 1 | 1 | 1 | 0 | 1 | 1 | 0 | 8 |
| De Almeida_2021 | 1 | 1 | 1 | 1 | 0 | 1 | 1 | 0 | 1 | 1 | 0 | 8 |
| Fourman_2021 | 1 | 1 | 1 | 1 | 1 | 1 | 1 | 0 | 0 | 1 | 1 | 9 |
| Guaraldi_2008 | 1 | 1 | 1 | 1 | 0 | 1 | 1 | 0 | 0 | 0 | 0 | 6 |
| Han_2023 | 1 | 1 | 1 | 1 | 1 | 1 | 1 | 1 | 0 | 1 | 1 | 10 |
| Ingiliz_2008 | 1 | 1 | 1 | 1 | 1 | 1 | 1 | 0 | 0 | 1 | 0 | 8 |
| Jongraksak_2021 | 1 | 1 | 1 | 1 | 0 | 1 | 1 | 0 | 1 | 1 | 0 | 8 |
| Kaplan_2020 | 1 | 1 | 1 | 1 | 0 | 1 | 1 | 0 | 1 | 1 | 0 | 8 |
| Kirkegaard-Klitbo_2020 | 1 | 1 | 1 | 1 | 1 | 1 | 1 | 1 | 1 | 1 | 0 | 10 |
| Lallukka-Brück_2020 | 1 | 1 | 1 | 1 | 1 | 1 | 1 | 0 | 0 | 1 | 1 | 9 |
| Liu_2021 | 1 | 1 | 1 | 1 | 0 | 1 | 1 | 0 | 1 | 1 | 1 | 9 |
| Lemoine_2017 | 1 | 1 | 1 | 1 | 0 | 1 | 1 | 0 | 0 | 0 | 0 | 6 |
| Lemoine_2022 | 1 | 1 | 1 | 1 | 1 | 1 | 1 | 0 | 1 | 1 | 0 | 9 |
| Lombardi_2016 | 1 | 1 | 1 | 1 | 0 | 1 | 1 | 0 | 0 | 0 | 0 | 6 |
| Lombardi_2017 | 1 | 1 | 1 | 1 | 1 | 1 | 1 | 0 | 0 | 0 | 1 | 8 |
| Lui_2016 | 1 | 1 |  | 11 | 1 | 1 | 1 | 1 | 0 | 0 | 1 | 8 |
| Maurice_2020 | 1 | 1 | 1 | 1 | 1 | 1 | 1 | 0 | 1 | 1 | 0 | 9 |
| Milic_2020 | 1 | 1 | 1 | 1 | 1 | 1 | 1 | 0 | 1 | 0 | 0 | 8 |
| Mohr_2018 | 1 | 1 | 0 | 1 | 0 | 1 | 1 | 0 | 0 | 0 | 0 | 5 |
| Morse_2015 | 1 | 1 | 1 | 1 | 1 | 1 | 1 | 0 | 0 | 1 | 1 | 9 |
| Nishijima_2014 | 1 | 1 | 1 | 1 | 0 | 1 | 1 | 0 | 0 | 1 | 0 | 7 |
| Pezzini_2021 | 1 | 1 | 1 | 1 | 0 | 1 | 1 | 0 | 1 | 1 | 0 | 8 |
| Prat_2019 | 1 | 1 | 1 | 1 | 0 | 1 | 1 | 0 | 1 | 1 | 0 | 8 |
| Price_2017 | 1 | 1 | 1 | 1 | 1 | 1 | 1 | 0 | 1 | 1 | 1 | 10 |
| Price_2019 | 1 | 1 | 1 | 1 | 1 | 1 | 1 | 0 | 1 | 0 | 0 | 8 |
| Riebensahm_2022 | 1 | 1 | 1 | 1 | 1 | 1 | 1 | 0 | 0 | 1 | 1 | 9 |
| Sim_2021 | 1 | 1 | 0 | 1 | 0 | 1 | 1 | 0 | 1 | 1 | 0 | 7 |
| Sebastiani_2022 | 1 | 1 | 1 | 1 | 1 | 1 | 1 | 0 | 1 | 1 | 0 | 9 |
| Shur_2016 | 1 | 1 | 1 | 1 | 0 | 1 | 1 | 1 | 0 | 0 | 1 | 8 |
| Villa_2021 | 1 | 1 | 1 | 1 | 1 | 1 | 1 | 0 | 1 | 1 | 0 | 9 |
| Vodkin_2015 | 1 | 1 | 1 | 1 | 1 | 1 | 1 | 0 | 0 | 1 | 0 | 8 |
| Vujanovic_2019 | 1 | 1 | 1 | 1 | 0 | 1 | 1 | 0 | 0 | 0 | 1 | 7 |
| Vuille-Lessard_2016 | 1 | 1 | 1 | 1 | 0 | 1 | 1 | 0 | 0 | 0 | 1 | 7 |
| Yanavich_2021 | 1 | 1 | 1 | 1 | 0 | 1 | 1 | 0 | 1 | 1 | 0 | 8 |
| Zizza_2017 | 1 | 1 | 1 | 1 | 1 | 1 | 1 | 0 | 0 | 0 | 0 | 7 |

**Table 5 Full Results of Subgroup Analyses and meta-regression**

| Outcome | Moderator | Coefficient | SE | 95%CI | t | F | p |
| --- | --- | --- | --- | --- | --- | --- | --- |
| Liver Steatosis | Age (n=34) | -0.02 | 0.02 | -0.07-0.02 | -1.00 | 0.99 | 0.33 |
|  | Female proportion (n=33) | -0.01 | 0.01 | -0.02-0.01 | -1.00 | 1.00 | 0.32 |
|  | Duration of HIV infection (n=20) | -0.04 | 0.06 | -0.09-0.16 | 0.59 | 0.35 | 0.56 |
|  | BMI (n=32) | 0.04 | 0.07 | -0.10-0.18 | 0.65 | 0.42 | 0.52 |
|  | Waist (n=14) | 0.02 | 0.04 | -0.06-0.10 | 0.51 | 0.26 | 0.62 |
|  | Overweight (n=12) | 0 | 0.02 | -0.04-0.03 | -0.08 | 0.01 | 0.94 |
|  | Metabolic syndrome (n=13) | 0 | 0.01 | -0.02-0.02 | -0.10 | 0.01 | 0.93 |
|  | Diabetes mellitus (n=22) | 0 | 0.02 | -0.05-0.05 | -0.07 | 0.01 | 0.94 |
|  | Hypertension (n=21) | -0.02 | 0.01 | -0.04-0.01 | -1.42 | 2.02 | 0.17 |
|  | HOMA-IR (n=11) | 0.18 | 0.17 | -0.21-0.58 | 1.06 | 1.12 | 0.32 |
|  | TG (n=30) | 0.01 | 0 | 0-0.02 | 2.15 | 4.61 | 0.04 |
|  | TC (n=26) | 0 | 0.01 | -0.02-0.02 | -0.33 | 0.11 | 0.75 |
|  | HDL (n=27) | -0.06 | 0.04 | -0.14-0.02 | -1.57 | 2.47 | 0.13 |
|  | LDL (n=21) | 0.01 | 0.01 | -0.02-0.04 | 0.74 | 0.55 | 0.47 |
|  | Glucose (n=17) | 0.06 | 0.03 | 0-0.13 | 2.06 | 4.23 | 0.06 |
|  | ALT (n=27) | 0.02 | 0.01 | 0-0.04 | 1.77 | 3.14 | 0.09 |
|  | AST (n=24) | 0.02 | 0.02 | -0.01-0.05 | 1.26 | 1.58 | 0.22 |
|  | GGT (n=11) | 0 | 0.01 | -0.02-0.02 | 0.28 | 0.08 | 0.78 |
|  | Duration of ART (n=23) | 0.08 | 0.04 | 0-0.17 | 2.03 | 4.12 | 0.06 |
|  | Current ART use (n=20) | -0.01 | 0.02 | -0.04-0.03 | -0.31 | 0.09 | 0.76 |
|  | Undetectable HIV RNA (n=24) | 0.01 | 0.01 | -0.02-0.04 | 0.70 | 0.49 | 0.49 |
|  | NRTI% (n=14) | 0 | 0.01 | -0.03-0.03 | -0.02 | 0 | 0.98 |
|  | PI% (n=18) | -0.01 | 0.01 | -0.03-0.02 | -0.65 | 0.43 | 0.52 |
|  | INSTI% (N=11) | 0.01 | 0.01 | -0.01-0.03 | 0.91 | 0.83 | 0.39 |
|  | Smoking (n=12) | -0.02 | 0.01 | -0.04-0.01 | -1.65 | 2.72 | 0.13 |
| Liver Fibrosis | Age (n=18) | -0.01 | 0.05 | -0.1-0.09 | -0.13 | 0.02 | 0.90 |
|  | Female proportion (n=18) | 0.01 | 0.02 | -0.02-0.04 | 0.54 | 0.30 | 0.59 |
|  | Duration of HIV infection (n=12) | -0.01 | 0.07 | -0.17-0.15 | -0.16 | 0.02 | 0.88 |
|  | BMI (n=18) | 0.21 | 0.12 | -0.03-0.46 | 1.84 | 3.40 | 0.08 |
|  | Waist (n=11) | -0.02 | 0.03 | -0.08-0.05 | -0.49 | 0.24 | 0.64 |
|  | Overweight (n=6) | -0.01 | 0.03 | -0.09-0.08 | -0.16 | 0.02 | 0.88 |
|  | Metabolic syndrome (n=8) | 0 | 0.03 | -0.07-0.06 | -0.11 | 0.01 | 0.92 |
|  | Diabetes mellitus (n=12) | 0.02 | 0.03 | -0.05-0.09 | 0.74 | 0.55 | 0.48 |
|  | Hypertension (n=10) | 0 | 0.03 | -0.07-0.08 | 0.11 | 0.01 | 0.91 |
|  | HOMA-IR (n=10) | 0.25 | 0.18 | -0.16-0.67 | 1.43 | 2.04 | 0.19 |
|  | TG (n=18) | 0.01 | 0.01 | -0.01-0.02 | 1.28 | 1.64 | 0.22 |
|  | TC (n=14) | 0.01 | 0.03 | -0.05-0.07 | 0.28 | 0.08 | 0.79 |
|  | HDL (n=15) | -0.08 | 0.09 | -0.27-0.12 | -0.84 | 0.71 | 0.41 |
|  | LDL (n=11) | -0.06 | 0.03 | -0.14-0.02 | -1.80 | 3.22 | 0.11 |
|  | Glucose (n=13) | 0.12 | 0.06 | -0.02-0.26 | 1.88 | 3.54 | 0.09 |
|  | ALT (n=12) | 0.01 | 0.01 | -0.01-0.04 | 1.27 | 1.62 | 0.23 |
|  | AST (n=11) | 0.02 | 0.02 | -0.02-0.07 | 1.11 | 1.23 | 0.30 |
|  | GGT (n=6) | 0.03 | 0.04 | -0.10-0.15 | 0.61 | 0.38 | 0.57 |
|  | Duration of ART (n=12) | -0.09 | 0.09 | -0.28-0.11 | -0.99 | 0.99 | 0.34 |
|  | Current ART use (n=9) | 0.03 | 0.09 | -0.18-0.24 | 0.34 | 0.11 | 0.74 |
|  | Undetectable HIV RNA (n=13) | -0.01 | 0.05 | -0.11-0.09 | -0.27 | 0.07 | 0.79 |
|  | NRTI% (n=5) | -0.04 | 0.04 | -0.15-0.07 | -1.17 | 1.38 | 0.33 |
|  | PI% (n=9) | -0.02 | 0.02 | -0.06-0.02 | -1.08 | 1.17 | 0.32 |
|  | INSTI% (N=7) | 0 | 0.01 | -0.03-0.02 | -0.19 | 0.03 | 0.86 |
|  | Smoking (n=6) | 0.01 | 0.02 | -0.05-0.06 | 0.30 | 0.09 | 0.78 |

| NAFLD | Current CD4^+^T |  |  |  | -4.42 | ＜0.01 | 1.51 | 0.22 |
| --- | --- | --- | --- | --- | --- | --- | --- | --- |
|  |  | ≤500/ul (n=4) | 0.38 | 0.35-0.41 | -7.21 | ＜0.01 |  |  |
|  |  | ＞500/ul (n=24) | 0.36 | 0.35-0.38 | -21.90 | ＜0.01 |  |  |
|  | Economy |  |  |  | -4.95 | ＜0.01 | 0.06 | 0.80 |
|  |  | Developed country (n=25) | 0.38 | 0.37-0.40 | -17.63 | ＜0.01 |  |  |
|  |  | Developing country (n=9) | 0.36 | 0.34-0.38 | -14.09 | ＜0.01 |  |  |
|  | Geographic regions |  |  |  | -8.42 | ＜0.01 | 2.36 | 0.50 |
|  |  | North America  (n=11) | 0.34 | 0.24-0.46 | -2.51 | 0.01 |  |  |
|  |  | Asia  (n=5) | 0.37 | 0.29-0.46 | -2.81 | 0.01 |  |  |
|  |  | Europe  (n=15) | 0.42 | 0.34-0.50 | -1.96 | 0.05 |  |  |
|  |  | South America  (n=3) | 0.35 | 0.32-0.39 | -7.43 | ＜0.01 |  |  |
|  | Diagnostic method |  |  |  | -5.85 | ＜0.01 | 5.97 | 0.31 |
|  |  | Transient elastography (TE) (n=15) | 0.41 | 0.36-0.46 | -3.25 | ＜0.01 |  |  |
|  |  | Computed tomography (CT) (n=6) | 0.22 | 0.11-0.39 | -3.06 | ＜0.01 |  |  |
|  |  | Magnetic resonance spectroscopy (H-MRS) (n=1) | 0.40 | 0.36-0.46 | -3.61 | ＜0.01 |  |  |
|  |  | Liver biopsy (LB) (n=3) | 0.46 | 0.27-0.67 | -0.34 | 0.73 |  |  |
|  |  | Ultrasound (US) (n=2) | 0.36 | 0.26-0.47 | -2.53 | 0.01 |  |  |
|  |  | More than 2 methods(n=7) | 0.45 | 0.32-0.58 | -0.80 | 0.42 |  |  |

| Liver Fibrosis | Current CD4^+^T |  |  |  | -4.39 | ＜0.01 | 0.38 | 0.54 |
| --- | --- | --- | --- | --- | --- | --- | --- | --- |
|  |  | ≤500/ul (n=2) | 0.33 | 0.13-0.62 | -1.16 | 0.25 |  |  |
|  |  | ＞500/ul (n=16) | 0.24 | 0.16-0.35 | -4.28 | ＜0.01 |  |  |
|  | Economy |  |  |  | -5.35 | ＜0.01 | 0.11 | 0.74 |
|  |  | Developed country (n=14) | 0.24 | 0.16-0.34 | -4.59 | ＜0.01 |  |  |
|  |  | Developing country (n=7) | 0.27 | 0.15-0.43 | -2.77 | ＜0.01 |  |  |
|  | geographic regions |  |  |  | -8.25 | ＜0.01 | 16.13 | ＜0.01 |
|  |  | North America  (n=6) | 0.30 | 0.20-0.43 | -2.98 | ＜0.01 |  |  |
|  |  | Asia  (n=5) | 0.13 | 0.08-0.19 | -7.76 | ＜0.01 |  |  |
|  |  | Europe  (n=9) | 0.23 | 0.13-0.35 | -3.87 | ＜0.01 |  |  |
|  |  | South America  (n=3) | 0.52 | 0.28-0.75 | 0.15 | 0.88 |  |  |
|  | Diagnostic method |  |  |  | -9.61 | ＜0.01 | 55.61 | ＜0.01 |
|  |  | Transient elastography (TE) (n=10) | 0.26 | 0.17-0.36 | -4.146 | ＜0.01 |  |  |
|  |  | Computed tomography (CT) (n=2) | 0.17 | 0.12-0.23 | -8.45 | ＜0.01 |  |  |
|  |  | Magnetic resonance spectroscopy (H-MRS) (n=1) | 0.21 | 0.15-0.28 | -6.60 | ＜0.01 |  |  |
|  |  | Liver biopsy (LB) (n=2) | 0.58 | 0.48-0.68 | 1.62 | 0.11 |  |  |
|  |  | Ultrasound (US) (n=0) | - | - | - | - |  |  |
|  |  | More than 2 methods(n=8) | 0.19 | 0.10-0.34 | -3.64 | ＜0.01 |  |  |


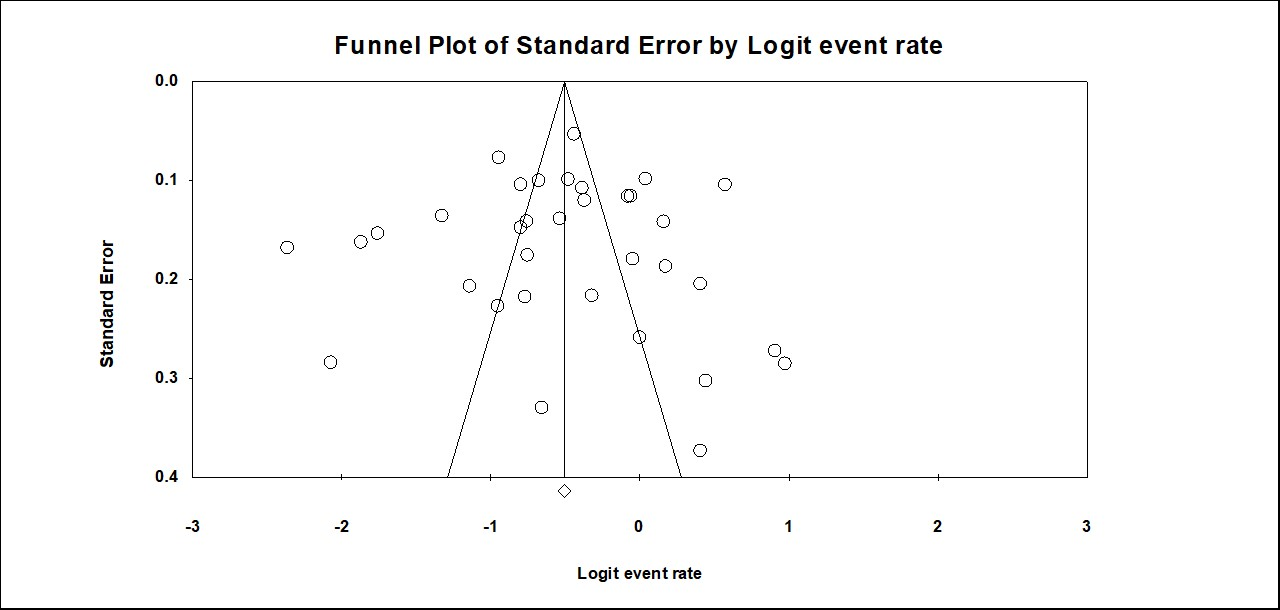
**Figure 1 Public bias of NAFLD prevalence**


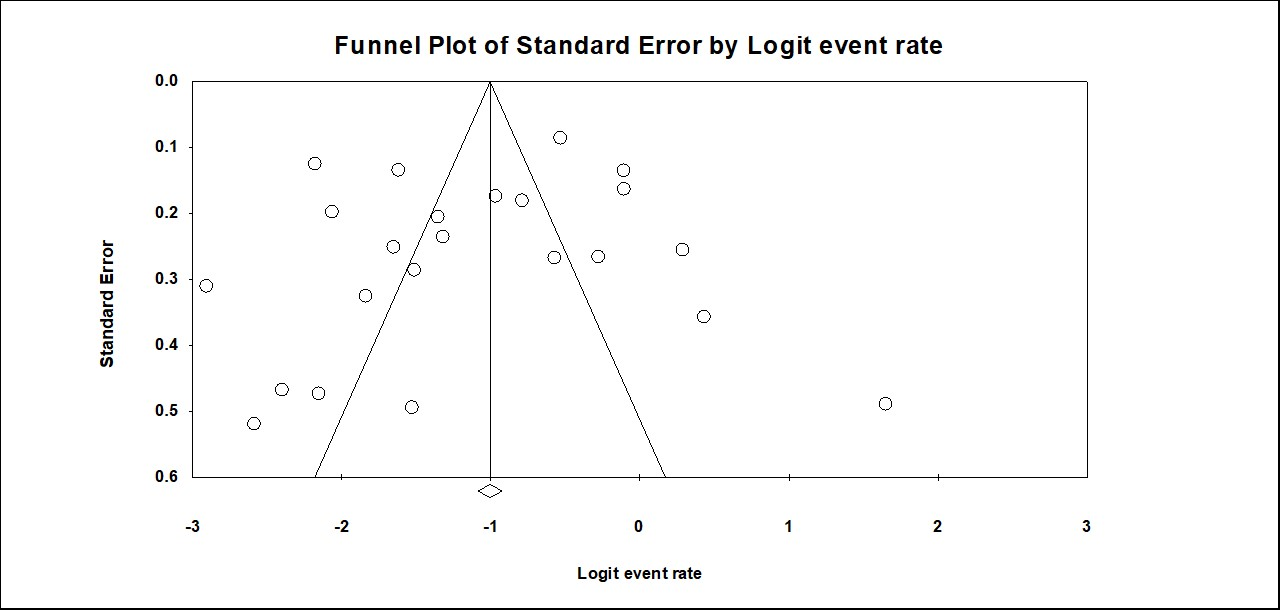
**Figure 2 Public bias of liver fibrosis prevalence**
